# Generational and Time Period Differences in American Adolescents’ Religious Orientation, 1966–2014

**DOI:** 10.1371/journal.pone.0121454

**Published:** 2015-05-11

**Authors:** Jean M. Twenge, Julie J. Exline, Joshua B. Grubbs, Ramya Sastry, W. Keith Campbell

**Affiliations:** 1 San Diego State University, San Diego, California, United States of America; 2 Case Western Reserve University, Cleveland, Ohio, United States of America; 3 University of Georgia, Athens, Georgia, United States of America; University of New South Wales, AUSTRALIA

## Abstract

In four large, nationally representative surveys (*N* = 11.2 million), American adolescents and emerging adults in the 2010s (Millennials) were significantly less religious than previous generations (Boomers, Generation X) at the same age. The data are from the Monitoring the Future studies of 12^th^ graders (1976–2013), 8^th^ and 10^th^ graders (1991–2013), and the American Freshman survey of entering college students (1966–2014). Although the majority of adolescents and emerging adults are still religiously involved, twice as many 12^th^ graders and college students, and 20%–40% more 8^th^ and 10^th^ graders, never attend religious services. Twice as many 12^th^ graders and entering college students in the 2010s (vs. the 1960s–70s) give their religious affiliation as “none,” as do 40%–50% more 8^th^ and 10^th^ graders. Recent birth cohorts report less approval of religious organizations, are less likely to say that religion is important in their lives, report being less spiritual, and spend less time praying or meditating. Thus, declines in religious orientation reach beyond affiliation to religious participation and religiosity, suggesting a movement toward secularism among a growing minority. The declines are larger among girls, Whites, lower-SES individuals, and in the Northeastern U.S., very small among Blacks, and non-existent among political conservatives. Religious affiliation is lower in years with more income inequality, higher median family income, higher materialism, more positive self-views, and lower social support. Overall, these results suggest that the lower religious orientation of Millennials is due to time period or generation, and not to age.

## Introduction

Are American adolescents any different in their religious orientation than they were a generation or two ago? Religious orientation includes belonging to a specific religion (religious affiliation), attending religious services, religious practices (such as prayer), religiosity (the importance of religion in life), spirituality, donating to religious organizations, and approving of religious organizations [1]. Adolescents’ religious orientation is important for several reasons. Religiosity is associated with a wide range of positive outcomes, including fewer risk behaviors [2], better social functioning [3], less substance abuse [4], and better physical health [5]. Relative to other adolescents, religious adolescents also report less depression, anxiety, and other psychiatric concerns [6] and display character strengths such as fidelity [7]. However, religion can also drive feelings of shame and guilt over certain behaviors [8, 9] and can be a source of struggle and distress [10].

In this paper, we examine time period and generational/birth cohort differences in American adolescents’ religious orientation, drawing from large, nationally representative surveys conducted over time (*N* = 11.2 million). These research designs examine participants of the same age at different points in time; thus, any changes must be due to time period or generation, but cannot be due to age or development. This method improves on one-time polls and surveys that cannot separate the effects of age and generation/time period. For example, if members of a younger generation appear less religious than members of an older generation in a one-time poll, that finding may be due to being young rather than to generational differences. Our use of four very large and parallel data sets suggests that the estimates of change will be reliable. Our goal is to present a comprehensive analysis of the over-time data on American adolescents’ religious orientation. The U.S. is an especially interesting culture to study in this regard, as its citizens are generally more religiously involved than those of other Western nations [11].

Theoretically, we conceptualize change over time and generations in attitudes, values, and personality traits as rooted in cultural change [12, 13]. That is, if individuals’ religious attitudes and behaviors change, we assume that this says something about changing cultural values. For example, the Mutual Constitution Model [12] describes cultures and individuals as influencing each other to create regional differences in culture; the same mechanism may operate as cultures differ over time periods. Generational differences are created when cultures change and new generations absorb that change when they are young, often during adolescence. Previous studies found significant generational and time period differences on a range of traits including decreasing empathy [14], greater personal vs. global fears [15], increasingly positive self-views [16], and decreasing trust in others and large institutions [17]. Overall, this research suggests that individualism has increased and social support has decreased. This pattern seems consistent with decreasing religious orientation, given that religion is often situated in institutions and focuses on more social values. Welzel [18] argues that nations have steadily increased in emancipation values, or the desire to be free of domination. If religion is perceived as a dominating force that restricts freedom and enforces social rules, this will be linked with a decline in religious involvement. Other studies have specifically linked individualistic traits (also known as agentic traits) to agnosticism, particularly in cultures with a relatively high rate of religious involvement [19].

Significant debate surrounds the question of trends in religious orientation among Americans. Several studies have revealed that the number of adults answering “none” to religious affiliation has increased, especially from the 1990s to the 2010s [20–23]. However, other studies suggest that religious service attendance, belief in God, and prayer have not changed or have even increased between the 1970s and the 2000s [24–26]. Thus there may be variation in the trends based on specific aspects of religiosity, demographic variables, and time periods examined.

In addition, research on trends in religious orientation in adolescent populations has been sparse, with few studies in the last 20 years (for example, Smith et al. [27] stopped with data from 1996). Examining 18- to 24-year-olds (young adults emerging from adolescence) in the General Social Survey (1972–2006), Smith and Snell [25] found only small changes in religious affiliation and service attendance, and no changes in frequency of prayer and belief in God. They concluded that emerging adults “have not since 1972 become dramatically less religious or more secular … if such a trend is indeed perceptible, it would seem to be weak and slight” (p. 99–100). Thus, it is unclear whether adolescents demonstrate the same decline in religious affiliation as adults do, and uncertain whether changes have occurred (in any age group) in other aspects of religious orientation such as religious service attendance, spirituality, prayer, religiosity, and attitudes toward religious organizations.

Thus, our question of interest was: Have American adolescents’ religious orientations changed in any meaningful way in the last few decades? We examine the religious orientation of Boomers (born 1946–1964, adolescents of the 1960s and 1970s), Generation X (born 1965–1981, adolescents of the 1980s and 1990s), and Millennials (born 1982–1999, adolescents during the 2000s and 2010s). Note that these generational cutoffs have little empirical basis; we use them only as convenient labels for those born in different decades. Also, we use the terms birth cohort and generation interchangeably, as both refer to being born during a certain period. Technically, however, a birth cohort is everyone born in one year, and a generation is everyone born in an often arbitrarily defined 20- to 30-year period.

We drew upon the Monitoring the Future (MtF) survey of American 8^th^, 10th, and 12th graders (*N* = 1.2 million) and the American Freshman (AF) survey of entering college students (*N* = 10 million). These surveys are all nationally representative, large, conducted each year for several decades, and inclusive of several questions on religious orientation. These analyses will provide a comprehensive picture of generational and time period differences in adolescents’ religious orientation disentangled from age and development, as age is held constant in each survey.

We also explore whether sex, race, socioeconomic status (SES), and political orientation moderate the trends over time; that is, do all adolescents, or only some groups, show generational/time period differences in their religious orientation? The rise in cultural individualism may have impacted some groups more than others. For example, given shifts away from traditional female roles, females may have been affected more than males, and Whites more than Blacks given the stronger religious tradition in the Black community [28, 29]. The shift toward individualism may have had less impact on political conservatives, who have traditionally emphasized social rules [19].

Finally, we examined correlations between mean religious affiliation and yearly social indicators, such as economic conditions (e.g., income inequality), social support (e.g., percent living alone), and cultural individualism (e.g., individualistic language, self-confidence, need for uniqueness). These analyses aimed to demonstrate which social factors covary with religious orientation. In other words, under what social conditions is religious affiliation high, and under what conditions is it low?

## Method

### Sample size and demographics

All datasets were available online, MtF as SPSS datafiles [30] and AF as data tables [31]. On each survey, we analyzed all items measuring religious orientation. All four surveys were designed to be nationally representative of the U.S. population at each educational level. Across all years, the 8^th^ (*n* = 338,912), 10^th^ (*n* = 306,148), and 12^th^ (*n* = 531,192) grade samples were 51% female, and the college students (*n* = 9,959,250) were 52% female. The 8^th^-12^th^ grade samples were 70% White, 12% Black, 10% Hispanic, and 4% Asian-American. Across all years ethnicity was measured (1971–2014), the college samples were approximately 80% White, 10% Black, 5% Hispanic, and 5% Asian-American.

### Sampling and measures

#### MtF 12th grade survey

MtF samples middle and high schools across the United States that have been chosen to represent a cross-section of the U.S. population (see http://www.monitoringthefuture.org). The participation rate of schools is between 66% and 80%, and the student participation rate is between 79% and 83% [30]. About 15,000 students in each grade (8^th^, 10^th^, 12^th^) are sampled each year in the spring. Some questions are only asked of subsamples.

The MtF survey of 12^th^ graders asked six questions on religion and religious organizations 1976–2013. They are: 1. “What is your religious preference?” Eighteen choices were offered, including “none.” (Atheist and agnostic categories were not included). These responses are not included in the datafiles due to privacy concerns; thus we obtained the percentage of “nones” in each year from the MtF annual data tables. In addition, this question was not asked of respondents in California after 1997. 2. “How often do you attend religious services?” Choices (coded 1–4) were “never,” “rarely,” “once or twice a month,” and “about once a week or more.” 3. “How important is religion in your life?” Choices (coded 1–4) were “not important,” “a little important,” “pretty important,” and “very important.” These 3 questions were not asked of respondents in California after 1997. 4. “How good or bad a job is being done for the country as a whole by … Churches and religious organizations?” Choices (coded 1–5) were “very poor,” “poor,” “fair,” “good,” “very good,” and “no opinion.” We excluded “no opinion” responses. 5. “Do you think the following organizations should have more influence, less influence, or about the same influence as they have now? How much influence should there be for … Churches and religious organizations?” Choices (coded 1–5) were “much less,” “less,” “same as now,” “more,” “much more,” and “no opinion.” We excluded “no opinion” responses. 6. “Are you likely to contribute to … Church or religious organizations?” Choices (coded 1–6) were “definitely not,” “probably not,” “don’t know,” “probably will,” “definitely will,” and “already have.” The last 3 questions were asked of subsamples and thus have lower *n*s. The data on religious preference are not included in the datafiles but are available at the aggregate level in the printed MtF databooks.

The 12^th^ grade survey also includes the respondents’ sex, race, region (Northeast, Midwest, South, and West), and parental education (which we used as a proxy for SES, with high = father attended college vs. low = father did not attend college; parental income and occupation are not assessed, and mother’s education is an unreliable indicator of SES in samples from earlier decades). Until 2000, the race question included only White and Black; thus we were only able to examine these two groups. We also examined political orientation by comparing those identifying as political liberals, moderates, and conservatives.

#### MtF 8th and 10th grade surveys

The 8^th^ and 10^th^ grade surveys, administered 1991–2013, asked questions on religious preference, attendance at religious services, and the importance of religion in life identical to those in the 12^th^ grade survey. The item on religious preference is not included in the datafiles, so we obtained this data by year from the survey administrators.

#### The American Freshman survey

AF surveyed a nationally representative sample of entering first-year students at four-year colleges or universities since 1966 [31]. We used the aggregated data with individual-level standard deviations [13, 16]. AF asked eight questions about religion and spirituality, including: 1. “Your religious preference” or “current religious preference,” including “None,” asked 1966–2014. 2. “Your father’s religious preference,” asked 1973–2014. 3. “Your mother’s religious preference,” asked 1970–2014. 4. “Attended a religious service … in the past year.” The percentage who answered “yes” is reported; thus we subtracted it from 100 to obtain the number who had not done so. This item was asked in 1966, 1968–1971, 1978–79, and 1981–2014. 5. “Rate yourself on each of the following traits compared with the average person your age. … Spirituality.” The percentage who chose “Highest 10%” or “Above average” is reported. Asked 1996–2014, except 2004. 6. “Clergy” under “Your probable career/occupation.” Asked 1966–2014, except 1973–75. 7. “For the activities below, indicate which ones you did during the past year … discussed religion.” 8. How much time “did you spend” on “Prayer/meditation,” with the choices “None,” “Less than one hour,” “1 to 2 hours,” “3 to 5 hours,” “6 to 10 hours,” 11 to 15 hours,” “16 to 20 hours,” and “over 20 hours” (coded 0–7) and asked 1996–2005.

#### Social indicators

We gathered annual statistics on economic factors, individualism, and social support, from publicly available sources, previous research, and the MtF and AF databases. Both MtF and AF include measures of self-confidence: MtF asks students to rate themselves as above or below average on intellectual ability and school ability, and AF asks if they are above or below average in intellectual self-confidence and social self-confidence. These, along with the measures of self-rated leadership ability and drive to achieve, are positive self-views consistent with individualism. For high school students, the measure of materialism was the importance of “having a lot of money;” for college students, it was those agreeing that “becoming very well-off financially” is important. Uncommon names, an indicator of need for uniqueness, are from the Social Security Administration database of names; we used boys’ names, as they change more consistently over time [32]. Individualistic words and phrases are from the Google Books database [33]. The percentage of 12^th^ graders expecting to earn a graduate or professional degree was an indicator of high expectations, as the actual percentage of people earning these degrees has not changed [34].

### Data analysis plan

Given the large size of the samples and the current movement away from statistical significance testing in psychology [35, 36], we focus primarily on means, standard deviations and effect sizes rather than significance tests in the tables. Also consistent with past practice using these datasets, we present data both graphically and statistically to provide a clear picture of the results [13]. We primarily use the tables to present means and the text to present percentages.

MtF data were weighted by the sampling weight; they were analyzed at the individual level with the exception of the religious preference item. Each age group (8^th^, 10^th^, 12^th^ graders) is analyzed separately so age is held constant as year (and thus birth cohort/generation) varies. We analyze the AF data at the group level because the individual-level data were not available for most years of the survey. For the group-level data, we calculated the individual-level SDs using SPSS. For example, if 60% of respondents agreed with an item in a particular year (and thus 40% did not), the individual-level *SD* of that sample is. 49. The use of the individual-level SD makes the effect sizes in individual-level and group-level data identical.

Data collected over time can be analyzed in many ways, including grouping by 20-year generation blocks, by decades, or by individual year. We determined that separating the data into 5-year intervals provided the best compromise between specificity and breadth. We report the effect sizes (*d*, or difference in terms of standard deviations) comparing the first group of years to the last, but we also 1) provide the means and SDs for 5-year intervals between these endpoints, so fluctuations at other times are apparent, and 2) provide figures with year-by-year results.

The analyses including the social indicators were performed at the group level. We focused on the 12^th^ grade and college samples, as they were collected over the most years. These analyses examine the correlation between the percentage of 12^th^ graders or college students professing no religious affiliation and the social indicators matched by year. These analyses cannot show causation, but they do demonstrate the cultural and social factors co-occurring with changes in the lack of religious affiliation.

## Results

**Fig 1:**
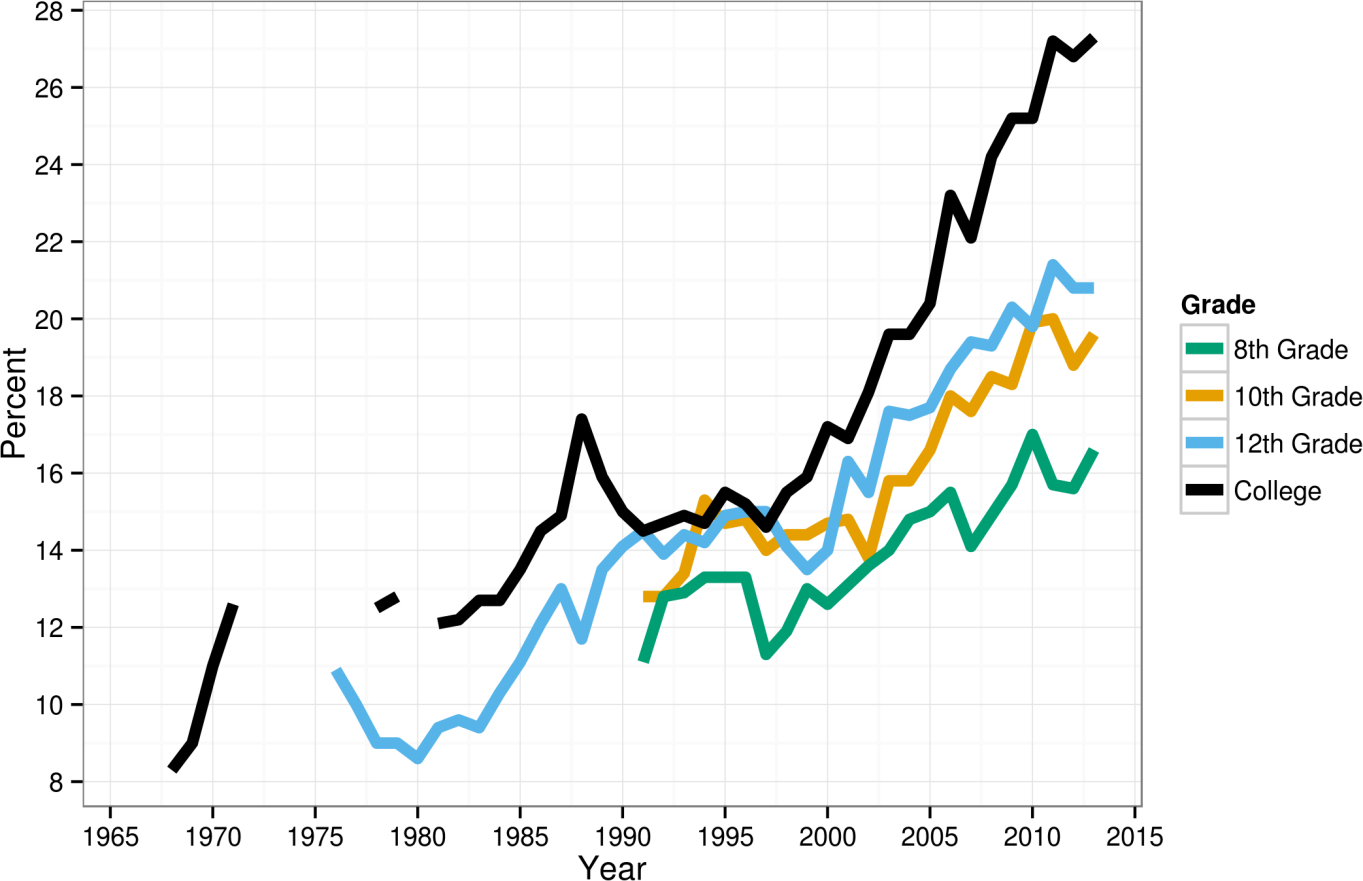
Percentage of American adolescents who never attend religious services, 1966–2013 (note: 1966 college data trimmed).

Recent cohorts of American adolescents are less religiously oriented than their predecessors, although the majority are still involved with religion (see Table 1). First, American adolescents are now less likely to attend religious services. Twice as many 12^th^ graders in 2010–13 reported “never” attending services (21%) compared to 1976–79 (10%). Compared to the early 1970s (12%), more than twice as many college students in the 2010s never attended services (27%). Similar, though smaller, declines (23% and 43%) appear among 8^th^ and 10^th^ graders between the early 1990s and the 2010s. Across all groups, the shift is most pronounced after 2000 as Millennials enter the samples, with the number not attending services increasing 50% for 12^th^ graders (from 14% to 21%), 33% for 10^th^ graders (15% to 20%), and 31% for 8^th^ graders (13% to 17%) between 2000 and 2013. The percentage attending services weekly has also declined steadily; while 40% of 12^th^ graders did so in 1976–79, only 30% did in 2010–13 (see Table 1 and Fig 1). For 10^th^ and 12^th^ graders, almost all of the decline in religious service attendance (*d*’s = -.13 and-.14) occurred between 2000 and 2013.

**Table 1:** Time period/generational differences in American adolescents’ religious orientation, 1976–2014.

	N	1966–69	70–74	75–79	80–84	85–89	90–94	95–99	00–04	05–09	10–14	*d*
**Religious service attendance (1–4 scale)**												
8^th^ graders	312,567	——-	——-	——-	——-	——-	2.91 (1.10)	2.92 (1.11)	2.85 (1.12)	2.85 (1.13)	2.81 (1.14)	-.09 (-.10)
10^th^ graders	294,603	——-	——-	——-	——-	——-	2.80 (1.10)	2.78 (1.10)	2.82 (1.12)	2.72 (1.14)	2.64 (1.14)	-.14(-.16)
12^th^ graders	521,137	——-	——-	2.87 (1.05)	2.86 (1.05)	2.70 (1.06)	2.66 (1.07)	2.67 (1.08)	2.67 (1.10)	2.59 (1.12)	2.55 (1.12)	-.30
**Never attend religious services**												
8^th^ graders	312,567	——-	——-	——-	——-	——-	13%	13%	14%	15%	16%	.09
10^th^ graders	294,603	——-	——-	——-	——-	——-	14%	14%	15%	18%	20%	.17
12^th^ graders	521,010	——-	——-	10%	9%	12%	14%	15%	16%	19%	21%	.31(.36)
College students	8,648,837	17%	12%	13%	12%	15%	15%	15%	18%	23%	27%	.24 (.41)
**Attend religious services once a week or more**												
8^th^ graders	312,567	——-	——-	——-	——-	——-	44%	44%	44%	42%	41%	-.06
10^th^ graders	294,603	——-	——-	——-	——-	——-	39%	38%	40%	37%	34%	-.10 (-.13)
12^th^ graders	521,010	——-	——-	40%	39%	33%	32%	32%	33%	31%	30%	-.22
**Religion “None”**												
8^th^ graders	338,912	——-	——-	——-	——-	——-	13%	13%	14%	16%	17%	.11
10^th^ graders	306,148	——-	——-	——-	——-	——-	13%	14%	15%	17%	20%	.19
12^th^ graders	531,192	——-	——-	10%	9%	12%	16%	17%	18%	20%	23%	.39 (.42)
College students	9,959,250	9%	13%	9%	8%	11%	13%	14%	17%	20%	25%	.43 (.47)
College students’ mothers	7,945,336	——-	3%	4%	5%	6%	6%	7%	8%	10%	12%	.36
College students’ fathers	7,797,873	——-	7%	7%	8%	10%	10%	11%	13%	15%	17%	.30
Difference between parents and students choosing “none”	7,797,873	——-	6%	4%	3%	4%	5%	5%	6%	7%	10%	.14 (.29)
**Churches/religious organizations doing a good job:** 12^th^ graders	88,338	——-	——-	3.73 (.97)	3.78 (.95)	3.63 (1.01)	3.61 (1.03)	3.68 (1.02)	3.63 (1.06)	3.56 (1.09)	3.50 (1.11)	-.22 (-.27)
**Churches/religious organizations should have more influence:** 12^th^ graders	91,800	——-	——-	3.51 (1.08)	3.45 (1.10)	3.21 (1.18)	3.33 (1.20)	3.43 (1.20)	3.35 (1.22)	3.20 (1.23)	3.09 (1.25)	-.37
**Willing to donate to churches/ religious organizations:** 12^th^ graders	104,509	——-	——-	4.35 (1.52)	4.34 (1.51)	4.12 (1.55)	4.06 (1.59)	4.07 (1.60)	3.91 (1.63)	3.83(1.65)	3.62 (1.67)	-.46
**Difference between donating to religion vs. fighting diseases:** 12^th^ graders	104,116	——-	——-	-.17 (1.59)	-.06 (1.58)	-.10 (1.65)	-.09 (1.70)	-.07 (1.70)	-.17 (1.69)	-.36 (1.74)	-.50 (1.78)	-.20 (-.27)
**Plan to become clergy:** College students	9,479,916	.93%	.83%	.61%	.42%	.34%	.36%	.46%	.44%	.34%	.40%	-.06
**Discussed religion:** College students	5,056,063	33%	30%	——-	——-	23%	24%	30%	30%	32%	31%	-.04 (.18)
**Consider self above average in spirituality:** College students	4,303,363	——-	——-	——-	——-	——-	——-	45%	40%	38%	36%	-.18
**Time spent in prayer or meditation (0–7 scale):** College students	2,671,439	——-	——-	——-	——-	——-	——-	1.21 (1.22)	1.16 (1.24)	1.10 (1.23)	——-	-.09
**Importance of religion in life (1–4 scale)**												
8^th^ graders	321,719	——-	——-	——-	——-	——-	2.78 (1.00)	2.86 (1.01)	2.88 (1.02)	2.82 (1.03)	2.74 (1.05)	-.04 (-.14)
10^th^ graders	294,791	——-	——-	——-	——-	——-	2.74 (1.02)	2.77 (1.03)	2.81 (1.05)	2.69 (1.06)	2.61 (1.07)	-.13 (-.19)
12^th^ graders	520,572	——-	——-	2.78 (.99)	2.82 (.98)	2.71 (1.00)	2.71 (1.04)	2.76 (1.05)	2.77 (1.06)	2.67 (1.08)	2.60 (1.11)	-.17 (-.21)

1. *d* = difference in standard deviations from the first period to the last. For variables with a non-linear change, the *d* from the lowest to the highest point is shown in parentheses.

2. For all *d*’s >. 03, the 95% confidence interval does not include zero.

**Fig 2:**
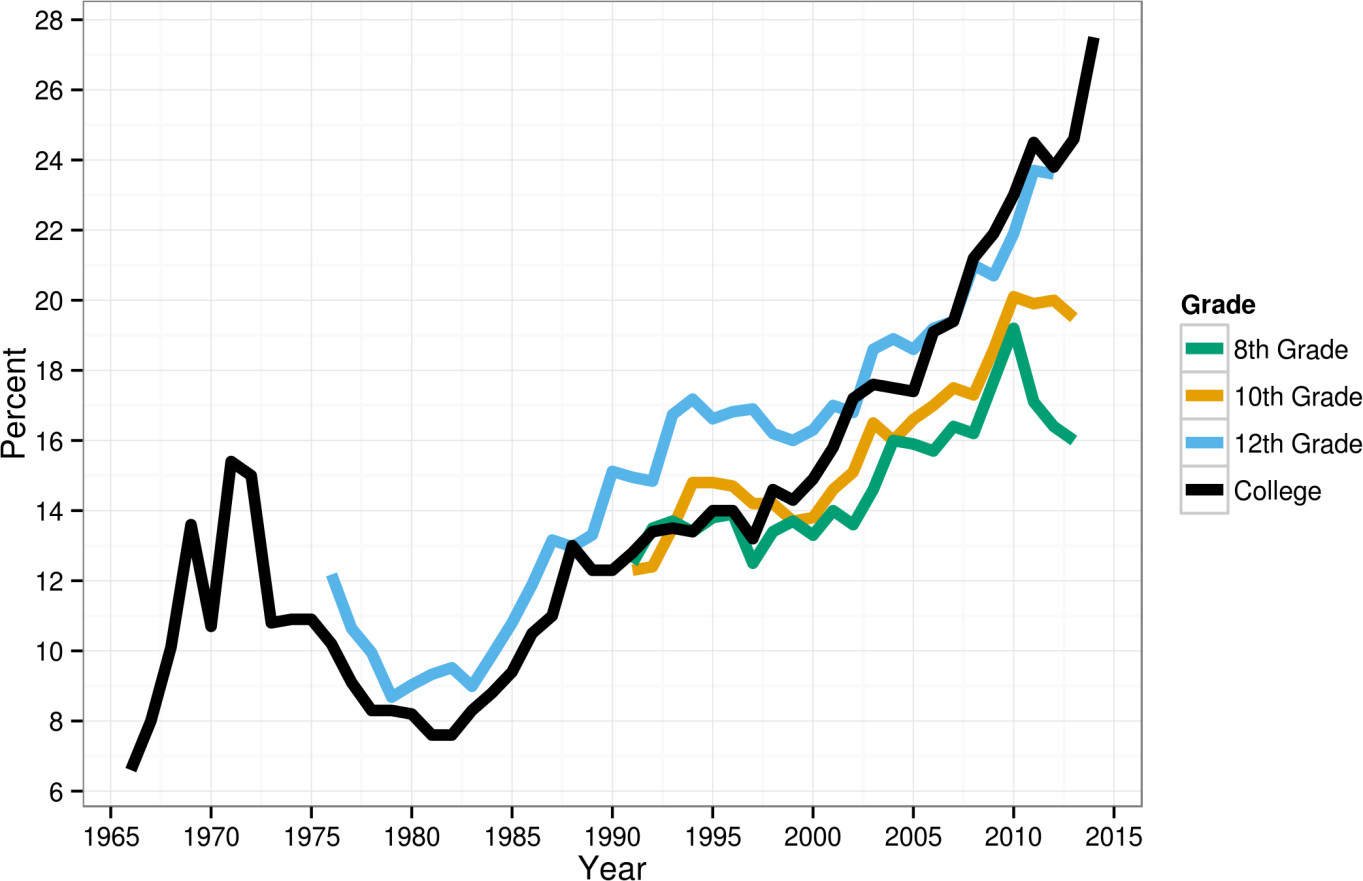
Percentage of American adolescents endorsing “none” for religious affiliation, 1966–2014.

More than twice as many recent 12^th^ graders chose “none” for their religious affiliation compared to the 1960s and 1970s, though the majority still choose a religious affiliation (see Fig 2). Thirty-eight percent more 8th graders and 53% more 10^th^ graders chose “none” as their religious preference in 2010–13 compared to 1991–94. The increase in religious “nones” was especially steep over the last decade. Between 2000 and 2010–13, 31% more 8^th^ graders (13% compared to 17%) professed no religious affiliation, as did 43% more 10^th^ graders (14% to 20%) and 50% more 12^th^ graders (16% to 24%). Three times as many college students in the 2010s (vs. the late 1960s) reported no religious affiliation, though the majority are still affiliated. In just the 13 years between 2000 and 2013, 87% more college students chose no religious affiliation (15% vs. 28%). Compared to the early 1970s, four times as many reported that their mother had no religious affiliation, and more than twice as many reported that their father had no religious affiliation. The gap between students’ affiliation and parents’ affiliation has grown (see Table 1); this suggests both that more students grew up without religion and that more are abandoning their parents’ religion by college entry.

Compared to those in the 1970s, 12^th^ graders in the 2010s are less likely to say that they believe that churches and religious organizations are doing a good job, less likely to say that they should have more influence, and less likely to donate to religious organizations (see Table 1). The decline in charitable donations is steeper for religious organizations than for other causes (see Table 1 for a comparison with charities fighting diseases). Entering college students in the 2010s were about half as likely as those in the late 1960s to say that they planned to enter the clergy (though this finding should be interpreted with caution given the low base rate). Differences in discussing religion were curvilinear, with fewer doing so during the late 1980s and 1990s, and few differences between the 1960s and the 2010s. Entering college students are now less likely to consider themselves above average in spirituality and less likely to pray or meditate (see Table 1). This suggests that recent generations of young Americans are less spiritual than their predecessors.

**Fig 3:**
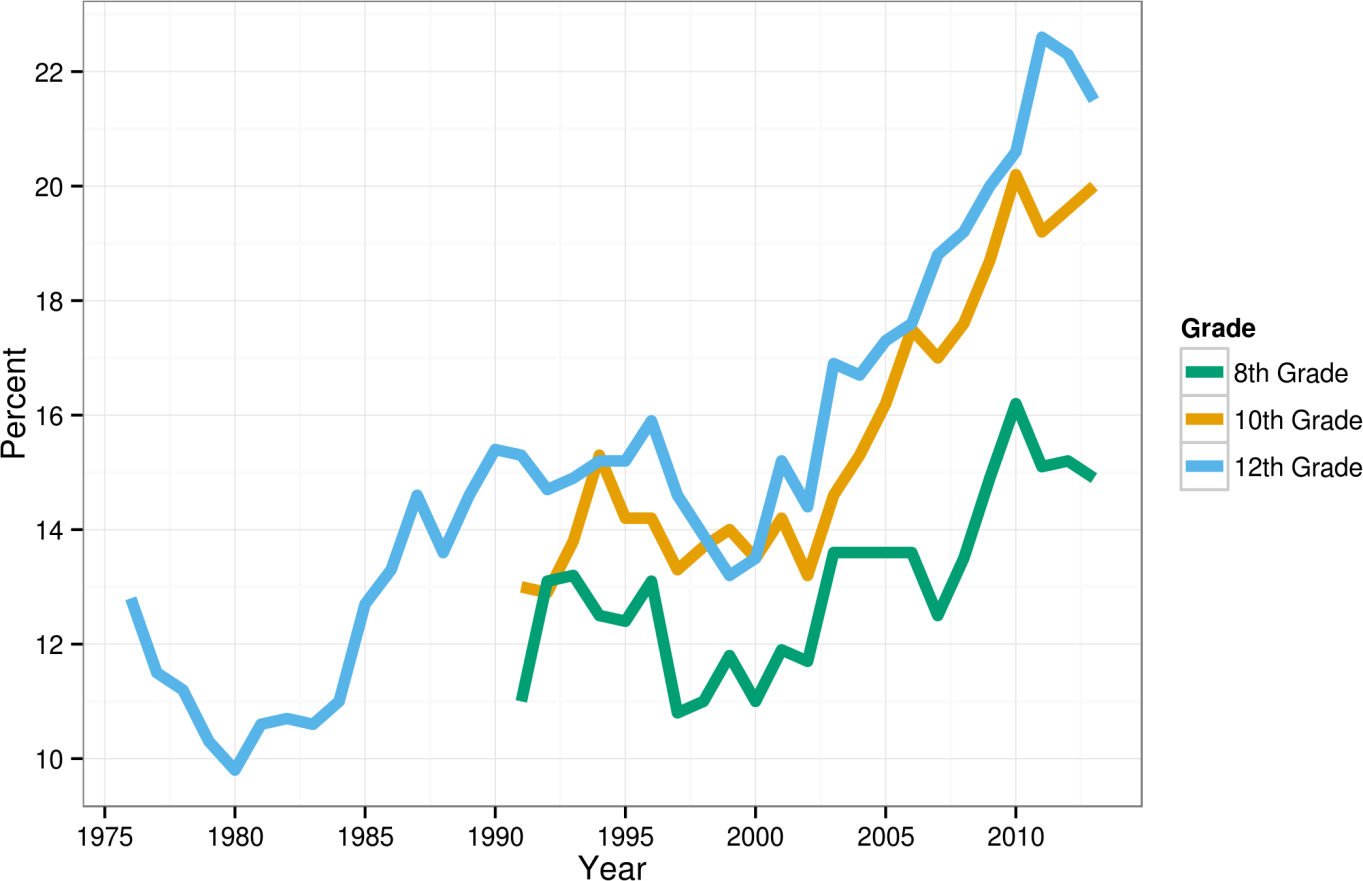
Percentage of American adolescents who say that religion is “not important” in their lives (low religiosity), 1976–2013.

Adolescents in the 2010s, especially 12^th^ graders, were less likely to say that religion is important in their lives (see Table 1 and Fig 3). In 2010–13, 22% said religion was “not important,” compared to 12% in 1976–79. Thus, although most still say that religion is at least somewhat important, 75% more 12^th^ graders said religion was “not important” to them. Much of this change occurred between 2000 and 2013, with those saying that religion was not important increasing 57% for 12^th^ graders (from 14% to 22%), 43% for 10^th^ graders (14% to 20%), and 36% for 8^th^ graders (11% to 15%). Most of the mean changes in the importance of religion in life for 10^th^ and 12^th^ graders (*d*’s = -16 and-.19) occurred after 2000.

These trends were moderated by sex, race, SES, U.S. region, and political orientation (see Table 2). Specifically, the decline in religious orientation is most pronounced among Whites, lower-SES individuals, girls, Northeasterners, and political liberals, and is smaller, though non-zero (*d* = -.07 for attendance and *d* = -.08 for importance of religion) among Black adolescents. Politically conservative adolescents did not differ in service attendance between the late 1970s and the 2010s, and actually increased in service attendance and the importance of religion between the late 1980s and the 2010s.

**Table 2:** Moderators of time period/generational differences in religious orientation, American 12^th^ graders, 1976–2013.

	N	75–79	80–84	85–89	90–94	95–99	00–04	05–09	10–13	*d*
**Religious service attendance (1–4 scale)**										
Males	246,850	2.76 (1.07)	2.77 (1.06)	2.61 (1.06)	2.58 (1.08)	2.60 (1.09)	2.59 (1.11)	2.53 (1.12)	2.49 (1.13)	-.25 (-.26)
Females	261,875	2.99 (1.03)	2.95 (1.03)	2.79 (1.05)	2.73 (1.06)	2.74 (1.06)	2.74 (1.09)	2.64 (1.11)	2.61 (1.11)	-.36
Black	64,718	2.93 (.96)	2.94 (.97)	2.91 (.98)	2.89 (.99)	2.92 (1.02)	2.96 (1.04)	2.93 (1.03)	2.86 (1.05)	-.07 (-.08)
White	377,892	2.88 (1.06)	2.87 (1.06)	2.68 (1.06)	2.63 (1.08)	2.64 (1.08)	2.62 (1.11)	2.54 (1.12)	2.49 (1.13)	-.36
Lower SES	238,609	2.84 (1.05)	2.82 (1.04)	2.65 (1.05)	2.57 (1.06)	2.58 (1.07)	2.55 (1.10)	2.49 (1.10)	2.43 (1.11)	-.38
Higher SES	245,896	2.94 (1.05)	2.94 (1.05)	2.77 (1.06)	2.75 (1.07)	2.76 (1.07)	2.79 (1.09)	2.70 (1.12)	2.69 (1.12)	-.23
Northeast	114,590	2.78 (1.07)	2.77 (1.06)	2.51 (1.05)	2.45 (1.05)	2.45 (1.05)	2.36 (1.08)	2.26 (1.06)	2.20 (1.06)	-.54
Midwest	154,585	2.92 (1.05)	2.90 (1.05)	2.75 (1.06)	2.66 (1.07)	2.63 (1.08)	2.62 (1.10)	2.62 (1.12)	2.57 (1.12)	-.33
South	193,583	2.98 (1.00)	3.00 (1.00)	2.86 (1.02)	2.81 (1.04)	2.82 (1.05)	2.87 (1.07)	2.73 (1.11)	2.71 (1.11)	-.26 (-.28)
West	58,380	2.71 (1.11)	2.64 (1.08)	2.55 (1.08)	2.55 (1.10)	2.64 (1.12)	——-	———	———	—-
Liberal	113,596	2.70 (1.06)	2.71 (1.05)	2.56 (1.05)	2.45 (1.04)	2.43 (1.05)	2.42 (1.09)	2.28 (1.08)	2.25 (1.09)	-.42 (-.43)
Moderate	146,305	2.98 (1.02)	2.94 (1.02)	2.78 (1.03)	2.75 (1.04)	2.77 (1.04)	2.78 (1.06)	2.69 (1.07)	2.66 (1.08)	-.31
Conservative	88,801	3.01 (1.03)	3.00 (1.03)	2.87 (1.05)	2.95 (1.06)	3.01 (1.06)	3.04 (1.07)	3.04 (1.07)	3.01 (1.08)	.00 (.16)
**Importance of religion in life (1–4 scale)**										
Males	246,475	2.63 (1.01)	2.68 (1.00)	2.59 (1.03)	2.60 (1.05)	2.65 (1.07)	2.64 (1.08)	2.55 (1.10)	2.49 (1.11)	-.13 (-.18)
Females	261,699	2.93 (.95)	2.95 (.94)	2.82 (.97)	2.82 (1.01)	2.87 (1.01)	2.89 (1.02)	2.78 (1.06)	2.71 (1.09)	-.22 (-.24)
Black	64,613	3.20 (.88)	3.23 (.86)	3.28 (.86)	3.26 (.89)	3.31 (.89)	3.31 (.90)	3.26 (.92)	3.13 (.99)	-.08 (-.16)
White	377,542	2.72 (.99)	2.75 (.98)	2.60 (1.00)	2.60 (1.03)	2.65 (1.04)	2.65 (1.06)	2.54 (1.08)	2.47 (1.10)	-.26 (-.28)
Lower SES	238.359	2.80 (.97)	2.84 (.96)	2.74 (.99)	2.72 (1.03)	2.77 (1.04)	2.74 (1.06)	2.66 (1.08)	2.56 (1.10)	-.24 (-.28)
Higher SES	245,645	2.74 (1.02)	2.79 (1.00)	2.66 (1.02)	2.70 (1.05)	2.75 (1.05)	2.79 (1.06)	2.68 (1.09)	2.64 (1.10)	-.09 (-.14)
Northeast	114,460	2.58 (.98)	2.63 (.97)	2.49 (1.01)	2.40 (1.03)	2.48 (1.05)	2.42 (1.06)	2.29 (1.07)	2.20 (1.07)	-.38 (-.43)
Midwest	154,407	2.72 (.96)	2.78 (.95)	2.63 (.98)	2.60 (1.01)	2.65 (1.04)	2.67 (1.05)	2.63 (1.07)	2.53 (1.09)	-.19 (-.25)
South	193,377	3.03 (.93)	3.07 (.91)	2.98 (.94)	3.00 (.97)	3.00 (.99)	3.05 (.99)	2.89 (1.05)	2.84 (1.08)	-.19 (-.23)
West	58,329	2.69 (1.07)	2.68 (1.06)	2.58 (1.05)	2.64 (1.08)	2.75 (1.10)	——-	———	———	——
Liberal	113,450	2.60 (1.03)	2.66 (1.01)	2.55 (1.04)	2.50 (1.06)	2.50 (1.07)	2.49 (1.09)	2.33 (1.10)	2.25 (1.11)	-.33 (-.39)
Moderate	146,184	2.84 (.95)	2.87 (.93)	2.74 (.97)	2.77 (.99)	2.83 (1.00)	2.86 (1.10)	2.73 (1.03)	2.70 (1.06)	-.14 (-.18)
Conservative	88,733	2.99 (.97)	2.99 (.97)	2.89 (1.00)	2.99 (1.01)	3.09 (1.00)	3.13 (.99)	3.10 (.99)	3.05 (1.03)	.06 (.16)

1. *d* = difference in standard deviations from the first period to the last. For variables with a non-linear change, the *d* from the lowest to the highest point is shown in parentheses.

2. For all *d*’s >. 05, the 95% confidence interval does not include zero.

3. These questions were not asked in California after 1996; thus, MtF does not report separated means for the Western region after that year.

The trend away from religious affiliation co-occurred with other social trends (see Table 3). We matched the annual percentage of 12^th^ graders and college students reporting no religious affiliation with annual statistics measuring economic factors, social support, and individualism. Although these analyses do not allow inferences about causation, they do give a view of the state of the society at times of high and low religious affiliation. More 12^th^ graders and college students reported no religious affiliation in years with higher median family income, more income inequality, higher materialism, less social support, and more individualism (such as higher self-confidence, a higher need for uniqueness, and more individualistic language in books). These analyses suggest that religious affiliation is low when the culture is high in individualism and low in social support.

**Table 3:** Correlations between the percentage of 12^th^ graders and college students choosing no religious affiliation and social indicators, matched by year.

	12^th^ grade	College
**Economic factors**		
Unemployment rate	-.19	.10
Median family income	.79***	.71***
Gini index of income inequality	.94***	.81***
**Individualism**		
Self-confidence (2 items)	.70***	.44**
Self-rated leadership ability	——	.63***
Self-rated drive to achieve	——	.79***
Materialism	.52**	.57***
Individualistic words	.72***	.53***
Individualistic phrases	.92***	.86***
High expectations (graduate degree)	.91***	——
Uncommon names (need for uniqueness)	.96***	.86***
**Social support**		
Percent living alone	.89***	.66***
Percent married	-.88***	-.75***
Birth rate	-.79***	-.75***

1. Correlations are weighted by sample size.

2. **p <. 01

***p <. 001

## Discussion

American adolescents in the 2010s (often called Millennials) are significantly less religiously oriented, on average, than their Boomer and Generation X predecessors were at the same age. However, the large majority still have at least some religious involvement. The generational/time period differences are notable, with some variables doubling or even quadrupling, and are most pronounced since 2000. Importantly, the declines extend to religious orientation outside of affiliation, showing decreases in religious service attendance and attitudes toward religious organizations. The declines also extend to the importance of religion, spirituality, and prayer, though these effects are both smaller and more limited. Thus, these results are not consistent with the idea that Americans are less religious but not less spiritual [37], but they are consistent with Smith and Denton’s [38] conclusion that today’s young Americans are not strong proponents of spirituality.

With the possible exceptions of Black Americans and political conservatives, these trends touched all major demographic groups, with larger declines for girls, low-SES individuals, Whites, and those living in the Northeast. Overall, the results suggest a movement toward secularism among a rapidly growing minority. As age is held constant in these datasets, the differences must be due to time period or generation and cannot be due to developmental stage. Thus, this data improves upon one-time polls finding that Millennials are less religious than GenX’ers and Boomers, a result that could have been due to age (perhaps younger people have always been less religious than older people). These analyses instead suggest a cultural change toward less religious involvement.

These conclusions differ from those of some previous research (e.g., Smith & Snell [25], who concluded that young adults were not significantly less religious). This difference may have occurred for several reasons. First, this study draws from more recent data (up to 2014), and the decrease in religious orientation is most pronounced during the last 10 years. Second, we draw from much larger samples. Third, the difference may lie in interpretation and analysis. For example, Smith and Snell [25] mention that the number of young adults claiming no religion rose from 14% in 1972–76 to 26% in 2004–06. The authors described this change as a 12% increase; however, a change from 14% to 26% might be better described as an 86% increase (26 – 14 = 12; 12/14 = 86% more choosing “none.”)

These results suggest that religious organizations are rapidly losing the youngest generation of Americans, known as Millennials. Most still have some religious involvement, but significantly more are not involved with religion at all. This is noteworthy because religion is often considered a major part of social identity [39] and transmits moral values through a sense of community [40]. Many people turn to religion as a meaning system [41] and a source of coping resources [42] and social support [43]. With religious orientation declining, fewer young people will have these resources. However, it is possible that other groups and activities (such as online activities) may take the place of religion, though it remains to be seen if they will provide the same benefits. Even religious adolescents may not reap the full benefits of religious involvement, as its links with health are stronger in societies with more religious tradition [44]. It is also possible that declines in religiosity could have some positive effects as well. For example, decreases in religious orientation may also correspond to decreases in shame and guilt that many religious individuals experience [8, 9]. Such decreases may also lead to fewer religious and spiritual struggles, which are also common among religious individuals [10]. In any case, however, it would be naïve to assume that these changes would be without effect. Given the central role that religious orientation has historically played in the psychosocial development of young people [25, 38], these shifts are not likely without consequence.

### Possible explanations for the decline in religious orientation

Why did these shifts occur? Several authors have argued that the primary cultural change in recent decades in the U.S. is an increase in individualism, characterized by more focus on the self and less on social rules [45–50]. The rise of individualism (focusing on the self rather than on others and society) may have led American adolescents away from religious orientation [51, 52]. The correlational analyses suggest that this may indeed be the case; religious involvement was low when indicators of individualism (such as more positive self-views, materialism, individualistic language, and need for uniqueness) were high. Religious involvement was also low when social support was low, and low social support is linked to high individualism [45].

This analysis cannot show causation, only correlation, but a connection between lower religious involvement and individualism has some theoretical and logical basis. First, religious orientation implies some level of commitment to a larger group or organization. In an individualistic society, people may become wary about making such commitments to organized groups; and indeed, such a reluctance to affiliate with organized groups has been on the rise in Western culture [13, 53, 54]. Second, belonging to a religious group may require assent with the group’s beliefs, opinions, and practices. This can create tension when differences in opinion arise between an individual and an organization [10]. These costs and compromises of group identification may be especially distasteful in a highly individualistic environment such as the modern-day U.S., which assigns high value to personal exploration, freedom of choice, and assertion of independent opinions.

Third, religious orientation usually involves some rule-following and submission to authority [40]. Consistent with this reasoning, religiosity shows negative associations with individualistic qualities such as hedonism, stimulation, and self-direction [55] (but see Gebauer, et al., [19] for a possible moderator of this effect). The tendency to resist any sort of external constraint could be especially pronounced among narcissists, whose sense of personal authority and entitlement makes them reluctant to submit to others [56]. Given that narcissism and overly positive self-views have increased [50, 57, 58] and respect for authority has decreased [59–60], these changes could also feed into lower religious participation. Fourth, religion often focuses on concerns outside of the self, such as helping others and serving God [61–62]. Potentially self-sacrificing virtues such as forgiveness, love, and gratitude are also highly valued within religious communities [63]. Thus, when people become deeply involved in religious faith, they may be committing to a value system that may bring some costs to the self – albeit with the hope of benefiting others. Finally, religion can involve a search for meaning, and this desire decreased markedly from the Baby Boomers to the Millennials [13].

Although the rise of individualism is one possible explanation for the decreasing interest in religion, other possibilities exist as well. Increasing religious pluralism in the U.S. could also result in the questioning or minimizing of all faiths (e.g., “the war on Christmas.”) However, that possibility does not explain the larger drops in religion in Europe, which generally has less religious diversity [64]. The increasing acknowledgment that religion is not consistent with scientific understanding of the universe may lead to a decrease in religion (e.g., the New Atheist Movement), but the conflict between scientific knowledge and many religious teachings goes back hundreds of years and thus cannot explain the recent timing of the decline. It is possible, however, that the reemergence of the science-religion conflict with debates about teaching creationism or intelligent design in U.S. schools, such as those in Kansas in 2005, pushed some young people away from religion. In addition, a generation of “digital natives” heavily involved in online activities might simply have been less interested in religious teachings. Another possibility is increasing high school graduation rates and college attendance, as more education is linked to lower religiosity [65]. However, in these data, adolescents whose fathers did not attend college actually showed a larger decline in religious orientation than those whose fathers attended college.

### Limitations and conclusions

One limitation of these analyses is that they cannot separate the effects of generation/birth cohort and time period. Thus, it is possible that Americans of all ages have become less religious over this time period. These designs do account for age and developmental effects, as age is held constant. Thus, Millennials’ lesser religious orientation is not due to their youth, but instead to their generation or the particular time period. Another limitation is that these datasets did not include in-depth measures of religiosity and spirituality. For example, we do not know whether declines in religious involvement were accompanied by struggles around religion [10] as opposed to simple indifference, or whether belief in God or an afterlife has changed. If American adolescents are still privately religious, they may still reap some of the benefits of a more involved religious orientation.

The MtF surveys stopped asking the questions on personal religious affiliation and beliefs in California after 1997. In a nationally representative sample of adults in 2008, 18% of California residents claimed no religion, compared to the national average of 15% [66]. With California 12% of the U.S. population, this probably lowered non-affiliation by about half of a percentage point. Thus, the MtF samples after 1997 most likely slightly underestimate the number of high school students with no religion and not attending religious services. If this is the case, the changes are actually larger than what is reported here.

In conclusion, survey results from 11.2 million American adolescents demonstrate a decline in religious orientation, especially after 2000. The trend appears among adolescents as young as 13 and suggests that Millennials are markedly less religious than Boomers and GenX’ers were at the same age. The majority are still religious, but a growing minority seem to embrace secularism, with the changes extending to spirituality and the importance of religion as well. Correlational analyses show that this decline occurred at the same time as increases in individualism and declines in social support. Clearly, this is a time of dramatic change in the religious landscape of the United States.
