# Correction: Generational and Time Period Differences in American Adolescents' Religious Orientation, 1966-2014

**DOI:** 10.1371/journal.pone.0221441

**Published:** 2019-08-15

**Authors:** Jean M. Twenge, Julie J. Exline, Joshua B. Grubbs, Ramya Sastry, W. Keith Campbell

The following information is missing from the Competing Interests statement:

JMT has consulted and received speaker honoraria from organizations (including public, private, and non-profit entities) based on her research exploring generational differences. These include honoraria from religiously affiliated colleges and universities including Calvin College, California Lutheran University, Geneva College, and the University of the Incarnate Word, though these honoraria were independent of the reported study.
